# Comprehensive Taste Profile Assessment of Underexplored Amino Acids and Protein Derivatives in Umami and Koku

**DOI:** 10.3390/foods15101826

**Published:** 2026-05-21

**Authors:** Manuel Ignacio López Martínez, Angelina Hopf, Ana Salvador, Fidel Toldrá, Ciarán Forde, Leticia Mora

**Affiliations:** 1 Instituto de Agroquímica y Tecnología de Alimentos (CSIC), Avenue Agustín Escardino 7, 46980 Paterna, Valencia, Spain; mi.lopez@iata.csic.es (M.I.L.M.); asalvador@iata.csic.es (A.S.); ftoldra@iata.csic.es (F.T.); 2 Laboratory of Food Chemistry, Wageningen University & Research, Bornse Weilanden 9, 6708 WG Wageningen, The Netherlands; angelina.hopf@wur.nl; 3 Sensory Science and Eating Behaviour, Wageningen University & Research, Stippeneng 4, 6708 WE Wageningen, The Netherlands; ciaran.forde@wur.nl

**Keywords:** amino acids, peptides, protein derivatives, taste, umami, koku

## Abstract

Taste strongly influences food acceptance and purchase intention. Beyond the five basic tastes, oral sensations such as astringency or koku modulate overall taste perception. Both umami and koku act as taste enhancers, increasing mouthfeel and savoriness. While the taste of most proteogenic amino acids is well established, non-proteogenic amino acids and related protein derivatives remain insufficiently characterized. This study analyzes the taste profile of seventeen underexplored amino acids and protein derivatives using the PredMol in silico tool and quantitative descriptive analysis (QDA), with particular emphasis on their umami and koku potential. In silico evaluation identified bitterness and sweetness as the predominant tastes and predicted carnosine, theanine, citrulline, and ornithine to have koku potential with values higher than 0.44. Principal Component Analysis of the QDA revealed that sweetness, bitterness, and sourness were the main drivers of sample differentiation. Ornithine, glutamine, citrulline, pyroglutamic acid, and theanine exhibited a positive dose–response in umami perception, with potential synergistic effects observed in the presence of 0.5 mmol/L IMP. Additionally, theanine, citrulline, and ornithine enhanced koku-related attributes, particularly aftertaste and continuity, in aqueous model solutions. Overall, these findings suggest that these compounds can have a taste influence in food products and potential to be used as taste enhancers.

## 1. Introduction

Taste, together with aroma and appearance, is a primary determinant of consumer acceptance and purchase intention in the food industry, making the identification and characterization of taste-active compounds a strategic priority for product development [1]. The human gustatory system detects five basic tastes (sweet, bitter, umami, salty, and sour) of which sweet, bitter, and umami are particularly influential in driving acceptance or rejection [2]. In addition, other oral sensations can have an impact on overall taste perception. As an example, astringency [3] and pungency [4] can have a negative effect on taste perception. In contrast, koku, a sensation that enhances mouthfulness, continuity, and aftertaste can contribute positively to the overall taste by increasing sweet, umami and salty taste perception [5].

While sugars, organic acids, and salts are classical contributors to taste, in protein-rich foods such as meat products, free amino acids and protein derivatives play a crucial role in shaping overall taste intensity and product appeal [2]. Although the taste of most proteinogenic amino acids have been widely studied [6], the sensory role of non-proteinogenic amino acids and protein derivatives present in food products remains poorly understood. In particular, their contribution to taste perception within complex food systems, as well as their potential involvement in modulating specific tastes, such as umami, has not been sufficiently characterized. This represents an important knowledge gap in understanding their overall impact on taste quality in foods.

Compounds such as ornithine, hydroxyproline or taurine have been reported in animal-derived foods [7,8,9], whereas high amounts of citrulline, betaine, and theanine have been reported in plant sources such as watermelon [10], beetroot [11], and green tea [12]. On the other hand, despite their limited presence in food products, protein derivatives such as β-hydroxy-β-methylbutyrate (HMB) [13], or 5-hydroxytryptophan [14], can be incorporated into functional foods and supplements due to their potential bioactive or ergogenic effects.

Umami taste and koku perception have technological relevance in the food industry both in terms of promoting product appeal and in supporting salt reduction. In fact, monosodium glutamate is a well stablished taste enhancer additive. Umami and koku substances can enhance the perception of desirable tastes, such as sweet or salty [15], promoting the development of healthier formulations with reduced salt or sugar content and improving the palatability in vulnerable populations, such as the elderly at risk of malnutrition [16]. In addition, koku compounds can increase the perception of viscosity without the need for added texture agents or fats, facilitating the development of reduced-fat food products without compromising the organoleptic properties [17].

In recent years, in silico approaches have gained increasing relevance in food science for the prediction of sensory-related properties of underexplored compounds. These tools enable rapid screening of molecular candidates and support the design of targeted sensory studies by reducing experimental burden [18]. However, as their predictions are based on computational models and training datasets, they may not fully capture the complexity of human sensory perception in real food systems. Therefore, experimental validation remains essential to confirm predicted sensory outcomes [19].

Therefore, the primary objective of this study was to comprehensively characterize the taste profile of seventeen commercially available amino acids and protein derivatives (citrulline, ornithine, glutamine, pyroglutamic acid, carnosine, theanine, HMB, norvaline, betaine, taurine, β-alanine, γ-aminobutyric acid (GABA), creatine, L-carnitine, hydroxyproline, hydroxytryptophan, and cystine) using a combined in silico and sensory approach, focusing on their potential capacity to exert umami taste and koku perception. These compounds remain largely underexplored from a sensory perspective, with some (e.g., norvaline and hydroxytryptophan) being evaluated for the first time to the best of our knowledge. These compounds were selected due to their notable presence in food products and their potential to exert a bioactive function and this evaluation aims to provide new insights into their contribution to overall taste perception and to support their potential application in the development of novel taste enhancers in food systems.

## 2. Materials and Methods

### 2.1. Broth Ingredients and Taste Compounds

Brown mushrooms (*Agaricus bisporus*), white onion (*Allium cepa*), spring onion (Allium fistulosum), garlic (*Allium sativum*), white radish (*Raphanus sativus* var. *longipinnatus*), unsalted crackers, bottled water, sucrose, sodium chloride (NaCl), and chilli pepper flakes (*Capsicum annuum*) were purchased in Albert Heijn (B.V., Oostzaan, The Netherlands) and Mercadona (Valencia, Spain). Monosodium glutamate (MSG) and inosine monosphosphate (IMP) were obtained from EPSA Aditivos Alimentarios (Valencia, Spain). Ornithine, taurine, creatine, citric acid and caffein were purchased from Buxtrade (Buxtehude, Germany). Glutamine, β-alanine, citrulline, γ-aminobutyric acid (GABA), L-carnitine, theanine, norvaline, β-hydroxy- β methylbutyrate (HMB), betaine, carnosine, pyroglutamic acid, reduced glutathione (GSH) and grape seed extract were purchased from Bulk Supplements (Nashville, TN, USA). Hydroxyproline, cystine and hydroxytryptophan were sourced from MarkNature (Fullerton, CA, USA). All ingredients were purchased in food-grade quality.

### 2.2. Study Design

The study design was performed in three phases:

The first part of the study consisted of in silico evaluation of the seventeen amino acids or protein derivatives (citrulline, ornithine, glutamine, pyroglutamic acid, carnosine, theanine, HMB, norvaline, betaine, taurine, β-alanine, GABA, creatine, L-carnitine, hydroxyproline, hydroxytryptophan and cystine) prior to the sensory analysis, to assess their potential taste profile and their koku potential.

The second part of the study involved quantitative descriptive sensory analysis of seventeen compounds. Data was collected with fourteen trained panelists who were asked to rate the intensity of the five basic tastes (sweet, salty, sour, bitter and umami), pungency and astringency of these compounds using a 100 mm unstructured scale anchored from “weak” to “strong”.

The third part of the study consisted in the umami and koku assessment of the six compounds with the most umami and koku potential of the previous part of the study. For this part twenty semi-trained panelists were asked to evaluate the umami, sweetness and bitterness of these six compounds at different concentrations using a general Labelled Magnitude Scale (gLMS), to determine their dose–response curves. For the koku assessment, the panelists were asked to evaluate several parameters related to koku perception in a gLMS, trying one concentration of these six compounds diluted in aqueous solution containing 0.5% NaCl (*w*/*v*) and 0.5% MSG (*w*/*v*) or mushroom broth.

### 2.3. In Silico Evaluation Procedure

Prior to sensory analysis, an in silico analysis was conducted on the seventeen amino acids or protein derivatives (citrulline, ornithine, glutamine, pyroglutamic acid, carnosine, theanine, HMB, norvaline, betaine, taurine, β-alanine, GABA, creatine, L-carnitine, hydroxyproline, hydroxytryptophan and cystine) using the PredMol machine-learning tool available in the TasteMeta platform (https://hwwlab.com/tastemeta/predmol) (Accessed on 25 October 2025) to assess their potential taste profile and their koku potential. PredMol is based on machine-learning algorithms that use molecule structures represented in SMILES format and extract their molecular fingerprints, such as Morgan-type fingerprints (MorganFP) to estimate the probability of specific sensory attributes [20]. MorganFP encodes molecular structures by representing the local atomic environments of each atom within a molecule as numerical features suitable for machine-learning models.

To carry out the in silico analysis, SMILES format chemical structures of the seventeen compounds were obtained from PubChem (https://pubchem.ncbi.nlm.nih.gov/), (Accessed on 25 October 2025), and subsequently analyzed using the algorithm of MorganFP-MLP. For each compound, probability scores ranging from 0 to 1 were obtained for six taste modalities and related sensations (sweet, salty, sour, bitter, koku, and astringency), where higher values indicate a greater likelihood of exhibiting the corresponding attribute.

### 2.4. Taste Profile Characterization

The second part of the study consisted of an evaluation of the overall taste profile of the seventeen compounds. Fourteen healthy adults were recruited from the research center “Instituto de Agroquímica y Tecnología de los Alimentos (IATA-CSIC)”. The recruitment criteria included being aged between 18 and 65 years old, non-smoker, non-pregnant and not having taste or smell dysfunction, allergies or intolerances. During these analyses, participants were instructed not to eat, consume coffee, or wear strong perfumes during the two hours preceding each tasting session. Panelists were required to read and sign the informed consent form prior to the start of the test and the ethical permission for the sensory analysis was granted by the CSIC institution (069/2025).

#### 2.4.1. Basic Taste Training

Firstly, the fourteen panelists were trained in basic taste recognition for one month. The training of the basic tastes was performed following the ISO 8586/2014 specifications with slight modifications [21]. The standard solutions used in this training were as follows: sucrose 5.76 g/L (sweet), NaCl 1.19 g/L (salty), citric acid 0.43 g/L (sour), caffeine 0.195 g/L (bitter), and monosodium glutamate 0.595 g/L (umami). In addition, the references for pungency and astringency were an aqueous infusion of chilli peppers (1 g/L) or grape seed extract (0.20 g/L), respectively. During the initial phase of training, panelists were presented with each reference solution together with its corresponding taste attribute. The analysis was conducted in five separate 30 min sessions, twice a week. In each session, panelists evaluated three different solutions, each corresponding to a different compound and they had to point out the tastes they identified. Samples were presented in a randomized order and coded with three-digit numbers. All sessions were carried out in a standardized tasting room with ten separate booths according to the ISO 8589/2010 Standard [22]. After the training sessions, all panelists were considered suitable for the study, as they demonstrated the ability to correctly discriminate the five basic tastes, as well as oral sensations such as astringency and pungency.

#### 2.4.2. Quantitative Descriptive Analysis

The QDA was performed according to Tanase et al. (2022) [9], with some adaptations, following the ISO 13299/2016 rule specifications [23].

For the sample preparation, each compound was diluted to 10, 20, 40 or 50 g/L in distilled water at 25 °C (Table 1) and aliquoted into 25 mL plastic white cups with a 3-digit code number. The concentrations used for sensory evaluation were determined through preliminary pre-tasting sessions (Table S1), in which levels were selected to ensure clear and consistent perception of all samples, allowing for reliable quantitative descriptive analysis (QDA) by the sensory panel. Panelists received coded samples, took a sip, swirled the liquid in the mouth for 30 s, and then spat it out and evaluated. The analysis of the intensity for each attribute (sweet, salty, sour, bitter, umami, pungent and astringent) was carried out on a 100 mm unstructured scale, where the left extreme represents 0 (no sensation), and the right end represents 10 (Strongest intensity). Between samples, panelists were forced to have a one-minute break, during which tap water was provided *ad libitum* along with unsalted crackers for palate cleansing.

### 2.5. Umami and Koku Substances Potential Assessment

The third part of the study involved the assessment of the six most promising compounds in terms of umami taste and koku perception. These compounds were selected based on either the highest perceived umami intensity in the QDA or the highest predicted koku scores from the PredMol tool. Twenty healthy adults were recruited from the Wageningen University Campus. The recruitment criteria included being aged between 18 and 65 years old, non-smoker, non-pregnant and not having taste or smell dysfunction, allergies or intolerances. During these analyses, participants were instructed not to eat, consume coffee, or wear strong perfumes during the two hours preceding each tasting session. Panelists read and signed the informed consent form prior to the start of the test and the ethical permission for the sensory analysis was granted by the research ethical committee (REC) of Wageningen University (Approval number 2025-263). The analysis consists of four 90 min sessions for one week. During the first session, a training session, panelists were introduced to the five basic tastes and familiarized with a general Labelled Magnitude Scale (gLMS). In the other three sessions, the umami and koku potential of each taste compound was assessed in the broth samples. Panelists received the samples in a randomized order and all samples were coded with three-digit numbers. All sessions were conducted in a sensory room at 20 °C, using individual booths.

#### 2.5.1. Training and gLMS Familiarization

The training in the five basic tastes was carried out as described in Section 2.4.1. Additionally, panelists were introduced to the koku perception. Therefore, there were two cups, one containing an aqueous solution with 5 g/L NaCl and 5 g/L MSG, and the other with the same aqueous solution spiked with 1.5 g/L GSH.

First, both were presented as non-koku and koku, respectively. Subsequently, two 3-digit coded cups were given, and the panelists were asked to select the koku sample.

After the training session, all panelists were considered suitable for the study, as they demonstrated the ability to correctly discriminate the five basic tastes, as well as koku perception.

For the gLMS familiarization, a gLMS partitioned by descriptors (no sensation (0), barely detectable (1.5), weak (6), moderate (17), strong (35), very strong (52) and strongest imaginable perception (100)) was used. In this scale, each panelist was asked to rank several imaginary sensations based on approaches reported elsewhere [24], such as how painful it is when you bite your tongue or how sweet is the candy floss.

#### 2.5.2. Umami Dose–Response Evaluation

The umami dose–response intensity evaluation was conducted following the sweetness protocol described by Wee et al. (2018), with adaptations for umami evaluation [24].

For sample preparation, each compound was diluted in water to obtain six concentrations following a 0.25 logarithmic increment series (Table 1) for the assessment of umami intensity. In addition, a separate set was prepared in the presence of 0.5 mmol/L IMP (0.174 g/L), according to Kawai et al. (2002), to evaluate potential synergistic effects with 5′-ribonucleotides [25]. One of the intermediate concentrations was duplicated and used as a warm-up sample at the beginning of each set to minimize first-order effects. Concentration levels were selected based on preliminary pre-tasting sessions (Table S2). During the evaluation, participants assessed umami, sweetness, and bitterness intensities using the generalized Labeled Magnitude Scale (gLMS). These attributes were included due to their potential interaction with umami perception. Each compound set consisted of eight samples: one water control, six concentration levels, and one warm-up sample. Sample orders within each set, as well as set presentation, were randomized using a Williams Latin square design, a balanced randomization method ensuring that each sample appears once in every position and follows every other sample an equal number of times across participants, thereby minimizing order and carryover effects. Panelists were instructed to take the coded sample, sip and swirl it in the mouth for 30 s, and then evaluate the intensity of each attribute before swallowing. A 30 s interstimulus interval was enforced between samples, during which panelists rinsed their mouth with water and consumed unsalted crackers ad libitum. Data were collected using EyeQuestion software (version 5.11.2, Logic8, The Netherlands).

#### 2.5.3. Koku Taste Assessment

##### Aqueous Model Solution Preparation

Aqueous model solution was prepared, mixing 4 L of bottled water with 20 g NaCl and 20 g MSG. For the koku training, 2 L of this solution were mixed with 3 g of GSH to have the spiked solution. Both solutions were prepared on the same day as the sensory evaluation.

#### Mushroom Broth Preparation

The mushroom broth was prepared following the beef broth from Brouwer et al. (2024) with some adaptations [26]. The broth consisted of 1.54 kg brown mushrooms, 317 g white onion, 233 g spring onion, 75 g garlic, and 280 g of white radish that were added to 7 L of bottled water in a pot. The mixture was heated to boil and simmered for 60 min at low heat. The broth was then cooled to 25 °C and strained through a double-layered cheesecloth to remove the vegetables. The broth was kept in the freezer at −18 °C and defrosted at 4°C for 24 h the day before the sensory session. For the sensory session, 1 L of mushroom broth was mixed with 1 L of tap water, 10 g NaCl and 10 g MSG, and stored at 4° C until use. All steps during sample preparation were performed under hygienic conditions in a food-grade room with food grade materials.

##### Training

For the koku training, panelists tried two cups, one of which was the aqueous model solution, and the other was the same solution spiked with GSH. First, both were presented as non-koku and koku, respectively. Subsequently two 3-digit code cups were given, and panelists were asked to guess which was the koku sample. Thereafter, they were instructed about five different properties (mouthcoating, body thickness, astringency, continuity and aftertaste) related to koku perception, given their descriptions and some clear examples about them, to ensure their understanding. These descriptions were extracted from previous publications [26,27] and compiled in Table 2.

##### Test Procedure

For sample preparation, each compound was diluted in aqueous solution (5 g/L NaCl, 5 g/L MSG) or mushroom broth. Each concentration was decided by pre-tastings. A total of 16 different samples (Table 1) were given in two sets, with 8 samples in each one, to the panelists. Samples were tasted as described in Section 2.5.2. All data was collected using EyeQuestion software (EyeQuestion software, version 5.11.2, Logic8, The Netherlands).

#### 2.5.4. Individual Standardization of Scaling with Modality Matching

To reduce inter-individual variability in the use of the gLMS scale, an individual standardization procedure was applied following Low et al. (2017), with minor modifications [28]. This approach was used to improve the comparability of intensity ratings across participants by correcting idiosyncratic differences in scale usage, which is a known source of variability in sensory scaling data. Participants were asked to rate the heaviness of six bottles coded with a 3-digit number, with increasing weight following a 0.25 logarithmic scale (Bottle 1 = 53 g, Bottle 2 = 251 g, Bottle 3 = 499 g, Bottle 4 = 724 g, Bottle 5 = 897 g, Bottle 6 = 1127 g). Participants held each bottle in the palm of their non-dominant hand and evaluated its perceived heaviness. Bottle presentation was randomized. A significant correlation between overall taste ratings and mean heaviness ratings was observed (r = 0.542, *p* = 0.03), supporting the suitability of the modality-matching approach. For each participant, an individual standardization factor was calculated as the ratio between the group mean heaviness rating and the participant’s mean heaviness rating. This factor was then applied to all individual umami and koku intensity ratings. This procedure helps minimize the bias associated with differences in scale usage, thereby improving the reliability and comparability of results across participants.

### 2.6. Statistical Analysis and Molecular Structure Design

For statistical analysis, linear mixed models (LMMs) were selected by treating panelists as random effects, LMMs explicitly account for inter-individual variability and within-subject correlations, thereby reducing the influence of individual differences and improving the reliability of fixed effect estimates such as compound or concentration effects in sensory data [29]. In both the quantitative descriptive analysis (QDA) and koku assessment, the following model was fitted using lme4 package [30] for each sensory attribute (sweet, salty, sour, bitter, umami, astringency and pungent in QDA; body thickness, mouthcoating, astringency, continuity, and aftertaste in koku taste):

Attribute ~ Compound + (1|Panelist)

Where “Compound” was treated as a fixed factor and “Panelist” as a random factor. Based on these models, marginal means were estimated (emmeans) and used for comparison between the tested compounds and compound concentrations. Statistics on models and the corresponding estimated marginal means were performed using chi-square analysis of the variance (ANOVA) tests together with Tukey post hoc test. Emmeans were used to visualize koku profiles via spider plots. Furthermore, Principal Component Analysis (PCA) was performed on the QDA data to show the distributions of the different compounds according to the taste attributes evaluated. For the umami assessment, a more complex LMM was fitted using lme4 package [30]:

Umami ~ Solution × log(concentration) × Compound + (Bitterness × Sweetness) + (1|Panelist)

Where solution, concentration, compound, bitterness and sweetness were considered as fixed factors and panelist was considered as random factor.

A chi-square ANOVA was carried out to determine which of the factors had a significant effect on umami. Slopes and intercepts were calculated to construct dose–response curves (umami growth factors). All statistical analyses were performed in R Statistical Software Version 4.6.0 [31].

Molecular structures of the different compounds were designed using ACD/ChemSketch, version 2025 (Advanced Chemistry Development, Inc., ACD/Labs, Toronto, ON, Canada).

## 3. Results and Discussion

### 3.1. In Silico Evaluation of the Compounds

According to Table 3, the in silico analysis indicated that the evaluated compounds could be classified into different predominant taste or oral perception categories. Norvaline, cystine and glutamine were predicted to be sweet, while betaine, creatine, hydroxyproline, L-Carnitine, pyroglutamic acid and taurine were mainly bitter; and carnosine and citrulline were mostly associated with koku. The other compounds displayed more heterogeneous taste distributions. Ornithine showed bitter and koku potential, while theanine was associated with astringency and koku sensation.

In addition, HMB was predicted to be bittersweet and astringent and hydroxytryptophan was classified as bittersweet. GABA showed potential to be astringent and tasteless, whereas β-alanine has more probability to be sweet, astringent and tasteless.

Overall, these in silico predictions are generally consistent with previously published literature. In this sense, results from Table 3 indicate that bitterness and sweetness are the predominant taste modalities predicted for the evaluated compounds. These findings align well with previous vivo sensory studies by Kawai (2012) on proteinogenic amino acids [32] and Tanase et al. (2022) on non-proteinogenic amino acids [9], including several compounds also investigated in the present study, such as ornithine, taurine, and citrulline, suggesting good agreement with in vivo results. Free amino acids and protein derivatives are generally described to elicit complex taste profiles, particularly involving sweet, bitter, and umami sensations [33]. From a mechanistic perspective, these sensory outcomes can be explained by the ability of amino acids and their derivatives to interact with G protein-coupled taste receptors involved in sweet (TAS1R2/TAS1R3), bitter (TAS2R), and umami (TAS1R1/TAS1R3) taste, respectively. In contrast, taste receptors that are structurally conformed as channels, such as epithelial sodium channels (ENaC) for saltiness or proton-selective channel (OTOP1) for sourness, do not directly interact with these molecules, which explains the absence of saltiness and sourness in the in silico predictions [34]. These agreements between the in silico predictions and sensory results may be explained by the use of molecular fingerprints in the in silico tool which encode structural features of the compounds associated with their interaction with taste receptors, allowing the indirect capture structure–taste relationships [35].

Nevertheless, the in silico approach has several important limitations. First, the absence of umami as a predicted taste represents a major shortcoming, as umami is one of the three main taste modalities commonly associated with amino acids. Second, the model does not consider concentration, despite the fact that the taste of these molecules is generally dose-dependent, and sometimes can shift with concentration increasing [32]. Third, koku perception remains poorly defined in predictive tools. Koku has been described as a perception that enhances mouthfulness, continuity, and complexity of foods. It is associated with the activation of the calcium-sensing receptor (CaSR), particularly by γ-glutamyl di- and tripeptides [36]. In fact, PredMol tool was successfully used in the screening of koku-related peptides in dry cured ham [37]. However, there is limited evidence showing that single free amino acids produce direct koku perceptions. Regarding these limitations and considering the high koku-related in silico scores for carnosine, theanine, ornithine and citrulline, these compounds were selected for the koku assessment. All compounds were subsequently evaluated through quantitative descriptive analysis (QDA) to determine whether in silico predictions were in alignment with in vivo sensory perception by trained panelists.

### 3.2. Quantitative Descriptive Analysis

According to QDA results (Table 4), clear differences in taste perception were observed among the evaluated compounds. β-alanine and GABA were perceived as sour and pungent, showing relatively high sourness (4.87 and 5.61, respectively) and pungency intensities (4.12 and 7.64), with GABA presenting the highest pungency among all compounds. β-alanine also exhibited moderate sweetness (2.98), partially aligning with in-silico predictions. Bitterness was one of the most prominent attributes, with ornithine, creatine and hydroxytryptophan showing high bitterness intensities (7.04, 5.36 and 7.30, respectively), while HMB presented the highest bitterness score overall (7.96) together with marked astringency (4.31), in agreement with in silico results. Taurine showed a mixed profile, combining moderate bitterness (4.25) and sourness (4.98). Several compounds, including betaine, carnosine, citrulline, cystine and norvaline, exhibited intermediate sweetness (3.44–6.62) combined with moderate to high bitterness (3.55–6.81), supporting their bittersweet character and agreeing with in silico predictions. In contrast, hydroxyproline showed the highest sweetness intensity (8.29), partially consistent with in silico results.

Sourness was particularly pronounced in pyroglutamic acid (9.75), which showed significantly higher values than all other compounds, and was also associated with elevated umami (5.00) and astringency (5.62), differing from in silico predictions.

Despite umami taste not being predicted in silico, glutamine and theanine exhibited notable umami perception (4.95 and 6.47, respectively), with theanine showing the highest umami intensity among all samples. In contrast, aligning with in silico results, none of the compounds were described as salty. Overall, bitterness, sourness and umami were the dominant sensory attributes, whereas sweetness showed greater variability and astringency contributed to the differentiation of specific compounds.

**Figure 1:**
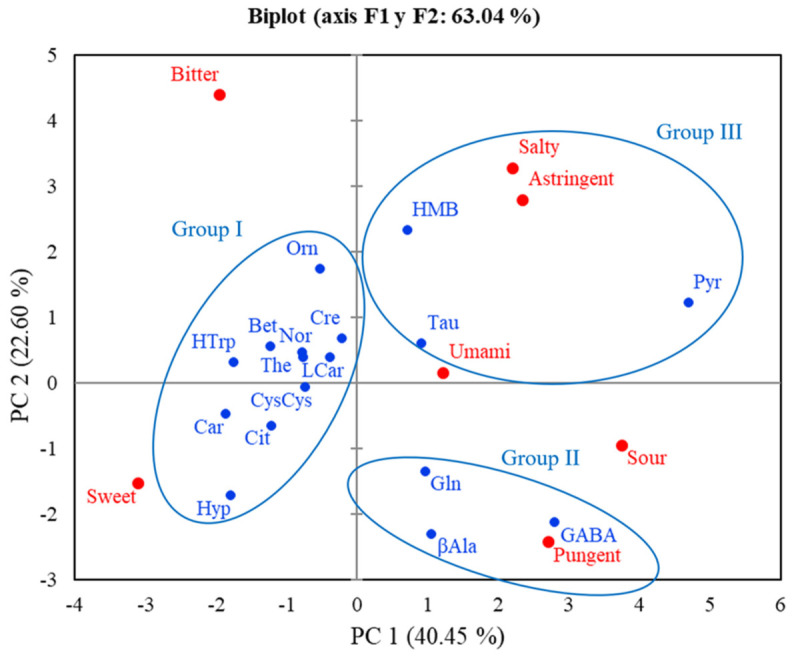
Principal component analysis of estimated marginal means (emmeans) for the results obtained during the quantitative descriptive analysis (QDA) of the seventeen compounds.

Principal component analysis (PCA) of the QDA data (Figure 1) showed that PC1 compiles 40.45% of the variance and PC2 accounted for 22.60%, explaining 63.0 5% of the overall variability. PC1 represented the main sensory gradient, separating compounds associated with sweetness and bitterness (negative values) from those characterized by sourness and pungency (positive values). PC2 provided additional discrimination, mainly driven by astringency and bitterness (positive values) in contrast to sweetness, sourness and pungency (negative values).

Based on this distribution, three main clusters were identified. Group I, located on the negative side of PC1, comprised compounds such as Hyp, Car, Cit, CysCys, LCar, Cre, The, Bet, Nor, HTrp and Orn, and was primarily associated with sweetness and bitterness. Group II (β-Ala, GABA and Gln) was positioned in the positive region of PC1 and negative PC2, showing a strong association with sourness and sweetness and/or pungency. Finally Group III (HMB, Tau and Pyr) was located in the positive region of both PC1 and PC2, indicating a combined contribution of sourness and astringency, with astringency acting as a key differentiating attribute for this group.

Overall, the PCA demonstrates that the main source of variability among compounds is driven by the opposition between bittersweet and sour attributes, while astringency contributes as a secondary factor in the sensory differentiation.

The multimodal interaction among amino acids and protein derivatives with G protein-coupled taste receptors [33] often complicates the assignation of a single taste [34]. Previous investigations have systematically evaluated the taste perception of free amino acids and related protein derivatives, reporting findings that are consistent with this study. In this sense, Kawai et al., assessed nearly all proteogenic amino acids in both L- and D-enantiomeric forms [32]. As an example, glutamine was described as sweet, sour, and umami, in agreement with this study [32]. Similarly, Tanase et al. (2022) evaluated the taste profile of ornithine, citrulline, GABA, taurine, β-alanine, and creatine, reporting comparable results to the present study at similar concentrations [9]. These findings support the taste perception patterns in the present study.

The structure and physico-chemical properties of each compound could explain the observed taste profiles. In this sense, according to the activation mechanisms of G protein-coupled receptor (GPCRs), sweet taste is mainly associated with amino acids containing small, hydrophilic side chains capable of interacting with the heterodimeric sweet receptor TAS1R2/TAS1R3, such as alanine or glycine [32,33]. Bitter taste is generally linked to amino acids with larger or more hydrophobic side chains, such as tryptophan or leucine, whose bulky or aromatic substituents enhance TAS2R receptors’ affinity [38,39]. Umami taste is primarily attributed to glutamate which activates the heterodimeric receptor TAS1R1/TAS1R3 and preferentially recognizes amino acids with a similar structure to glutamate and peptides with amino acids with acidic (e.g., Glu, Asp) and certain basic (e.g., His, Arg, Lys) side chains [34].

**Figure 2:**
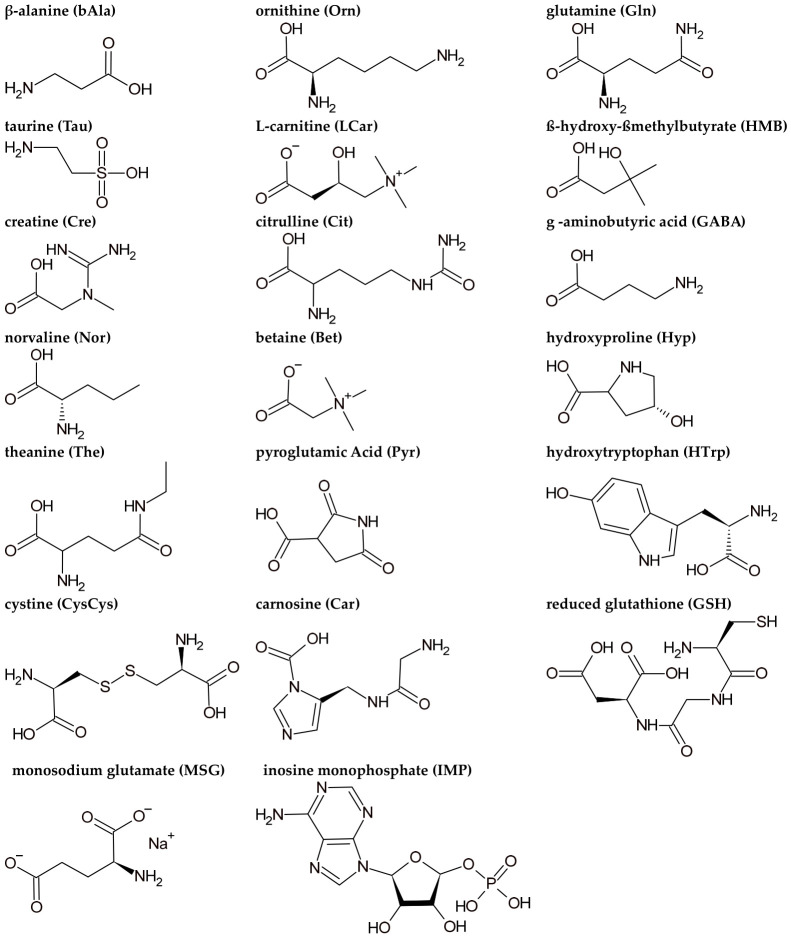
Structure designed using ACD/ChemSketch, version 2025 (Advanced Chemistry Development, Inc., ACD/Labs, Toronto, ON, Canada) of the tested compounds in the sensory analysis.

Regarding the structure of the compounds tested in this study (Figure 2), there are three tendencies that may explain their taste, which are (i) homology with a proteogenic amino acid, (ii) modification of a proteogenic amino acid structure, and (iii) the amino acid composition of the peptides.

Regarding homology, β-alanine and norvaline are isomers of alanine and valine, respectively. HMB is a derivative of leucine, maintaining its hydrophobic chain, responsible for the bitter taste [40]. Citrulline and ornithine are structurally similar to arginine [41], explaining their bitter or bittersweet taste. By contrast, pyroglutamic acid, glutamine, and theanine have similarities with the glutamate carbon skeleton, which might explain their potential umami taste. In fact, theanine was already reported that can elicit umami taste due to their interaction with the TAS1R1/TAS1R3 receptor [42].

Observing the modification of a proteogenic amino acid, the results of the study suggest that the addition of a hydroxyl group may enhance the sweet taste, according to hydroxyproline and hydroxytryptophan taste profiles. The modifications of glycine with methyl or guanidino groups, as it occurs in betaine and creatine, respectively, seemed also to enhance the bitterness perception.

Finally, the amino acid composition of a peptide sequence may explain their taste, since carnosine is a dipeptide composed of β-alanine (sweet) and histidine (bitter) [43], whereas cystine is a dimer of two cysteines (bittersweet) [44], suggesting that this amino acid composition explained their bittersweet taste.

Regarding sour taste, acidic or negatively charged groups (COO^−^ or SO_2_^−^) present in pyroglutamic acid or taurine could promote sourness, due to pH lowering and the generation of H^+^ ions that activate the sour taste receptor OTOP1 [9,34]. As the compounds in this study were dissolved in distilled water, there are no Na^+^ ions that can interact with amino acids or the ENaC receptor [45], explaining the absence of saltiness.

Regarding oral sensations, the perception of astringency perceived in HMB and pyroglutamic acid may be explained by their possible interaction with salivary proteins, promoting their precipitation, and producing the astringent perception [3]. Despite GABA being reported as pungent by Tanase et al., (2022), the mechanism of action remains unclear [9].

The taste of the compounds evaluated in the study can determine their influence in food products and their suitability to be added as functional ingredients. In this sense, hydroxyproline was determined to be extremely sweet at relatively low concentrations, suggesting their positive influence in food products and their potential use as an alternative sweetener. In this regard, collagen hydrolyzates with the presence of this amino acid were added to chocolate desserts, promoting their functionality without compromising palatability [46]. In contrast, other compounds, such as creatine or HMB, showed an excellent ergogenic potential for sport nutrition [13,47]. However, as these results showed, their potential bitterness can affect negatively their use as supplements or their addition to food products.

The second part of the study described the sensory profile of seventeen compounds, several for the first time (e.g, Norvaline, HMB). A key limitation is that all compounds were tested at only one concentration. Several amino acids and protein derivatives show dose-dependent taste responses; therefore, their sensory profiles may differ at other levels [32]. Due to the relevance in the food industry of umami in enhancing pleasant taste perceptions such as sweetness and saltiness, dose–response evaluations were conducted for the compounds with the highest umami ratings (glutamine, theanine, and pyroglutamic acid). Additionally, compounds predicted in silico to show strong koku activity (carnosine, ornithine, and citrulline) were also included due to the potential synergistic interactions between umami and koku molecules on their receptors [48].

### 3.3. Umami Taste Assessment

The Type II Wald chi-square tests of the linear mixed model (Figure S1) showed that umami perception was significantly affected by solution type (water vs. IMP), logarithmic concentration, and amino acid identity (*p* < 0.001). These results indicate that umami intensity is strongly dependent on both compound type and dose. In addition, several interaction effects were significant, including solution × concentration, solution × amino acid, and concentration × amino acid, indicating that the effect of dose on umami perception is not uniform but varies depending on both the compound and the presence of IMP. A significant three-way interaction (solution × concentration × amino acid) further confirms that the dose–response behavior of each amino acid is modulated by the solution condition. Bitterness did not show a significant main effect on umami perception, while sweetness had a significant positive contribution, suggesting a partial sensory cross-modal enhancement. These findings support a dose-dependent umami perception, consistent with previous reports evaluating the dose–response taste profile of amino acids with and without IMP, showing similar behaviors in substances such as glutamine or ornithine to the ones reported in this study [9,25,49]. The positive effect of IMP can be explained due to the synergistic effect produced when 5′-ribonucleotides (e.g., IMP, GMP, AMP) bind the TAS1R3 subunit while amino acids such as Asp and Glu bind the TAS1R1 one, stabilizing the umami receptor and enhancing the perception [16]. The positive contribution of sweetness may be explained by cross-modal or structural interaction through the TAS1R3 subunit, common in the umami (TAS1R1/TAS1R3) and the sweet receptor (TAS1R2/TAS1R3) [34]. Previous evidence indicating that some umami compounds can interact with bitter receptors and attenuate bitterness perception may account for the absence of a significant umami–bitterness interaction in the present study [50].

**Figure 3:**
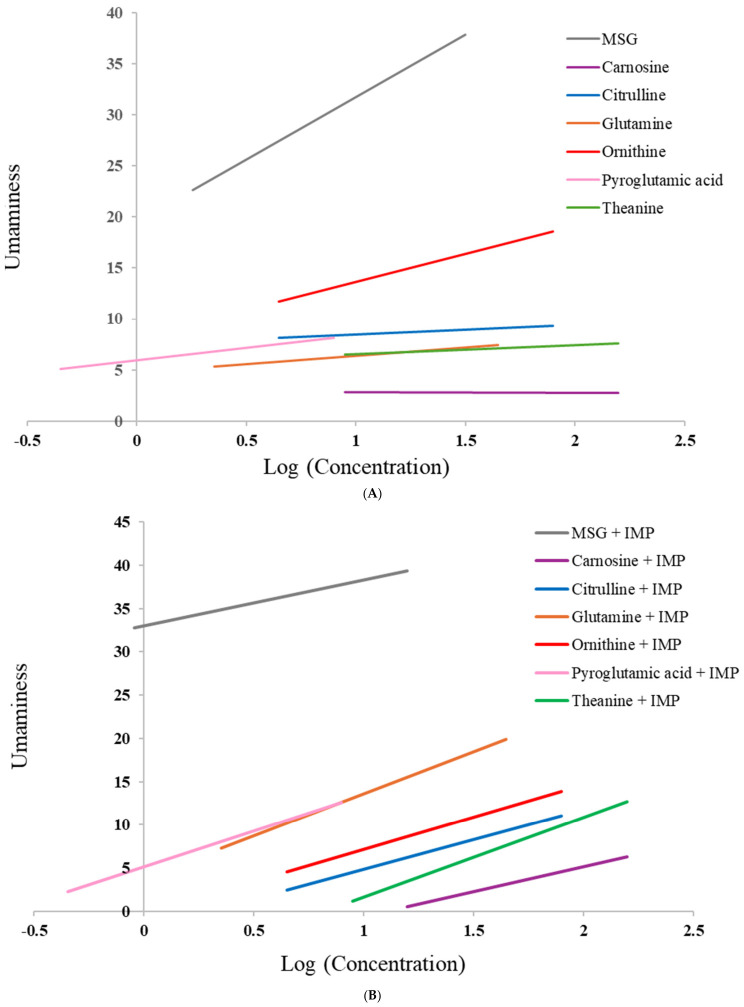
Dose–response umaminess curves obtained by the linear mixed model in water (**A**) or IMP 0.5 mmol/L solution (**B**) per compound (gray: monosodium glutamate (MSG), blue: citrulline, red: ornithine, pink: pyroglutamic acid, orange: glutamine, purple: carnosine, green: theanine).

Dose–response linear regressions obtained for the linear mixed model further supported these results. In water (Figure 3A), MSG produced the highest umami response, followed by ornithine. Glutamine, pyroglutamic acid, theanine, and citrulline showed positive modest dose-dependent umami enhancement, whereas carnosine showed a negative slope (Table S3), suggesting no umami potential at the tested concentrations.

In the presence of IMP (0.5 mmol/L), MSG remained the most intense stimulus, although with a reduced slope, possibly due to receptor saturation [51]. Glutamine, pyroglutamic acid, theanine, and carnosine exhibited enhanced positive slopes (Table S3) (Figure 3B) in the presence of IMP, suggesting synergistic interactions not previously reported for these compounds. Ornithine did not show an increased slope with IMP 0.5 mmol/L, contrary to earlier studies [9], which may be attributed to differences in tested concentrations, sensory methodologies (gLMS vs. VAS), or panel variability. Structurally, the similarity of theanine, pyroglutamic acid, and glutamine to the glutamate carbon skeleton may facilitate interaction with the umami receptor [34]. In addition, ornithine is derived from glutamate [52] and structurally related to citrulline in the urea cycle [41]. Although citrulline and ornithine did not show umami taste in water at tested concentrations in the second part of the study, their structural resemblance to glutamate (Figure 2), might explain their capacity to elicit umami taste. For carnosine, its peptide structure and potential interaction with sweet taste pathways may allow cross-activation or modulation of the umami receptor in the presence of IMP.

The combination of umami compounds with IMP is one of the current strategies in the development of food products with salt reduction has been reported to allow sodium reductions of up to 40% in certain formulations, while maintaining consumer acceptance [16,53]. Although the amino acids evaluated in this study did not exhibit a clear dose–response relationship for umami perception comparable to glutamate, their potential application as taste enhancers, either individually or in combination, warrants further investigation. Previous studies have shown that the combination of certain amino acids, such as leucine or valine, with monosodium glutamate (MSG) can enhance saltiness perception beyond that achieved by MSG alone [54]. Such synergistic effects could be exploited in the development of reduced-sodium food products, contributing to improved nutritional profiles and potentially reducing the risk of hypertension.

Overall, these findings suggest that umami perception is strongly dose-dependent and reveal previously underexplored synergistic effects between IMP and non-classical umami compounds. Sensory evaluation was conducted using semi-trained panelists. Although inter-individual variability was statistically controlled through the use of a linear mixed model and by bottle rating standardization, perceptual variability cannot be entirely excluded. In addition, IMP was tested at only one concentration (0.5 mmol/L), which restricts interpretation of the synergistic ranges. Future studies should explore a broader range of nucleotide and amino acid concentrations, to determine the optimal conditions for enhancing umami.

### 3.4. Koku Potential Assessment

**Figure 4:**
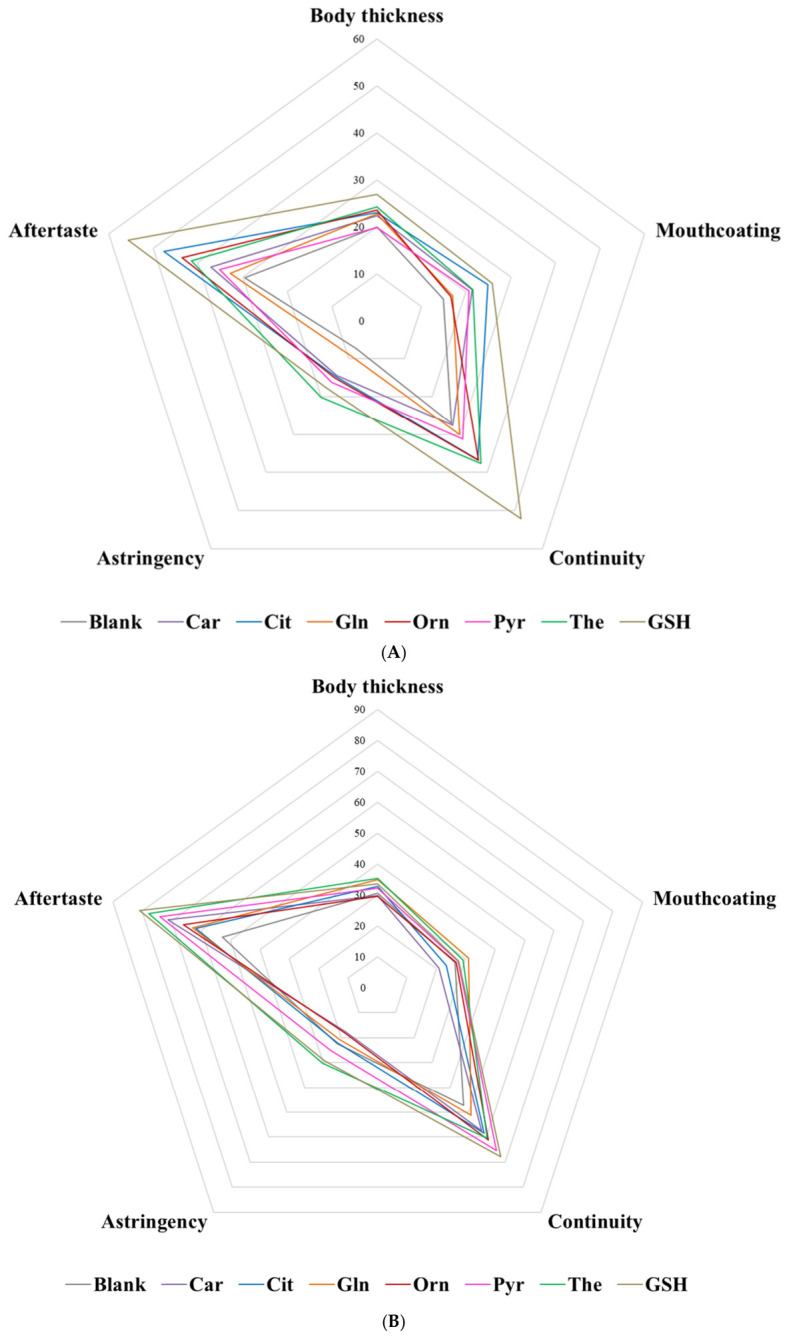

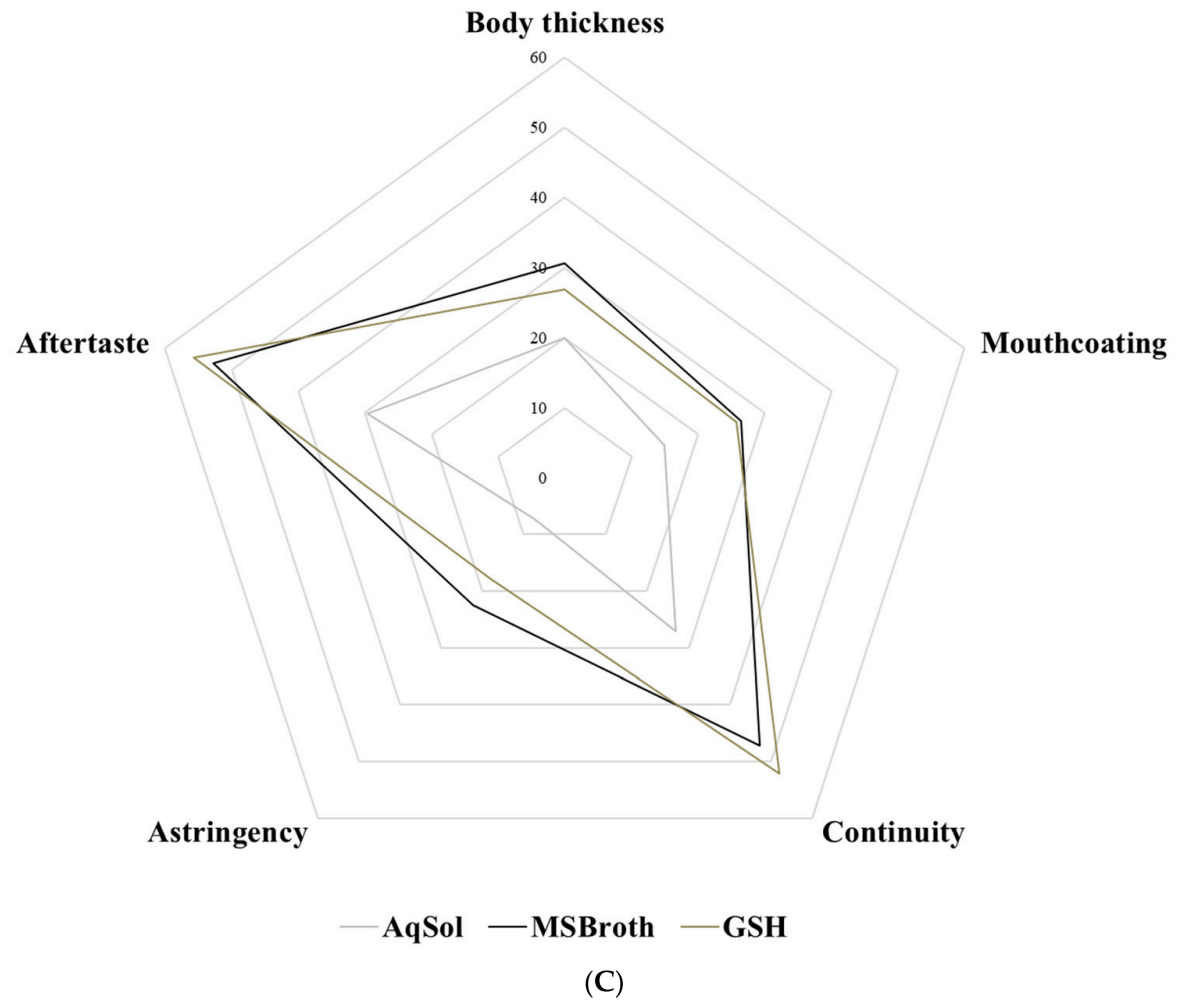
Spider plots showing the results of the koku assessment expressed as estimated marginal means (emmeans). (**A**) Aqueous model solution and (**B**) mushroom broth, with values presented per compound (gray: blank; blue: citrulline; red: ornithine; pink: pyroglutamic acid; orange: glutamine; purple: carnosine; green: theanine; brown: reduced glutathione) across the evaluated attributes (body thickness, mouthcoating, continuity, astringency, and aftertaste). (**C**) Comparison of koku assessment results for the blank in aqueous solution (gray line), reduced glutathione in aqueous solution (brown line), and the blank mushroom broth (black line), expressed as estimated marginal means (emmeans).

Figure 4A shows the estimated marginal means of the dissolved compounds in the aqueous model solution (water containing 5 g/L NaCl and 5 g/L MSG). At 1.5 g/L GSH exhibited the most pronounced enhancing effect across all evaluated sensory parameters. It showed the highest intensities in continuity, body thickness, mouthcoating and aftertaste, and was second in astringency, after theanine. After GSH, citrulline and theanine displayed the highest values for continuity and aftertaste, and together with ornithine the highest intensities in mouthcoating. In contrast, carnosine, pyroglutamic acid and glutamine showed responses closer to the blank control.

In mushroom broth (Figure 4B), differences between compounds were reduced compared to the aqueous model. GSH again exerted the strongest enhancing effect on most sensory attributes, but the relative distances between compounds were smaller than in model aqueous solution. After GSH, theanine showed the highest astringency, whereas pyroglutamic acid, theanine, ornithine, and carnosine exhibited higher values for continuity and aftertaste. In contrast, glutamine and citrulline produced responses closer to the blank in these parameters.

The results obtained from this part of the study are partially in agreement with the in silico results (Table 3), since theanine, citrulline and ornithine seemed to exert at these concentrations an enhancement in some koku-related parameters.

The reduced differences in the mushroom broth results may be attributed to its complex composition. It contains multiple taste-active constituents, including sulphur compounds, organic acids, 5′-ribonucleotides, free amino acids [55,56] glutathione [57], and koku peptides, such as EWVPVTK and EYPPLGR [58]. As a result, the mushroom broth showed comparable intensities to GSH in aqueous solution (Figure 4C).

GSH (γ-Glu-Cys-Gly) is a γ-glutamyl tripeptide well-known as a koku-related compound [36,58,59], so it was chosen as positive control for the enhancement of the koku-related parameters evaluated on this study. The relatively simple background of NaCl and monosodium glutamate allows koku-mediated enhancement of saltiness and umami to be more expressed.

Regarding theanine, Lu et al. (2022) observed that this amino acid may interact synergistically with some koku peptides, including glutathione and γ-Glu-Gln, thereby enhancing koku-related perception [12]. Such interactions may explain the effects observed in the mushroom broth. However, the results obtained from the aqueous model solution suggest that theanine may also contribute to the modulation of koku-related attributes such as continuity or aftertaste, although the mechanism of action remains unclear.

Pyroglutamic acid showed a weaker but similar effect to that of theanine in mushroom broth. Consequently, it may also be explained by synergistic interactions with compounds present in the mushroom broth.

Recently, it has been reported that ornithine can interact with the class C G protein-coupled receptor GPRC6A, which, similarly to CaSR, is associated with koku modulation [60]. This mechanism could explain its effects in continuity and aftertaste observed in the aqueous model solution. Its reduced effect on mushroom broth may be attributable to the presence of other compounds that can mask its function. In addition, this broth may also contain endogenous ornithine, naturally present in mushrooms [56]; therefore, small additions of ornithine may not be sensorially perceived.

The mechanism of citrulline on certain koku-related attributes in the aqueous model remains speculative. Its close structural relationship with ornithine and its metabolic interconversion within the urea cycle [41] suggest that citrulline could (i) activate by itself the GPRC6A receptor, or (ii) partially be converted to ornithine in solution.

These results suggest that, at tested concentrations, some compounds such as theanine, citrulline, ornithine, or pyroglutamic acid could enhance koku attributes. However, the complexity evaluation of the koku-related attributes, as well as the use of semi-trained panelists, remains a limitation and future studies should test different compound concentrations in different matrices to determine dose-dependent and matrix effects, since this perception generally occurs at low concentrations. Evaluation with and without nose clips should also be performed in the future, to analyze the influence of retro-nasal volatiles on these perceptions.

## 4. Conclusions

This study analyzes sensorially with QDA the overall taste profile of seventeen commercially available underexplored amino acids and protein derivatives, including HMB and norvaline, which were taste profiles that were assessed for the first time. Some of them, such as hydroxyproline or pyroglutamic acid, showed high taste intensity at low concentrations, suggesting their potential influence on taste in the food products where they are present. Ornithine, glutamine, citrulline, pyroglutamic acid, and theanine, showed positive dose-dependent umami responses. In addition, all of these umami-active compounds demonstrated synergistic interactions with IMP, supporting their potential role as taste enhancers. Koku-related effects were observed in both aqueous solution and mushroom broth, with ornithine, theanine, and pyroglutamic acid showing the most relevant contributions. In conclusion, these results identify specific amino acids and derivatives with strong taste-modulating properties and highlight their potential contribution to overall taste in food products and their potential use as alternative taste enhancers. Future studies should evaluate their inclusion in real food matrices and further investigate their mechanisms of action using techniques such as electronic tongue and molecular docking.

## Figures and Tables

**Table 1:** Compilation of the different concentrations and abbreviations of the compounds used in the sensory analysis.

Concentrations for the Quantitative Descriptive Analysis							
Compound	Taurine (Tau)	Citrulline (Cit)	Pyroglutamic Acid (Pyr)	Glutamine (Gln)	Theanine (The)	Carnosine (Car)	Ornithine (Orn)
Concentration (g/L)	50.00	20.00	10.00	20.00	40.00	40.00	20.00
Compound	HMB ^a^	norvaline (Nor)	hydroxyproline (Hyp)	creatine (Cre)	betaine (Bet)	GABA	L-carnitine (LCar)
Concentration (g/L)	20.00	20.00	10.00	10.00	20.00	40.00	50.00
Compound	β-alanine (βAla)	cystine (CysCys)	hydroxy- tryptophan (HTrp)				
Concentration (g/L)	40.00	10.00	10.00				
Concentrations for the umami potential assessment							
Compound	MSG	citrulline	pyroglutamic acid	glutamine	theanine	carnosine	ornithine
Concentration 1 (g/L)	1.80	4.45	0.45	2.25	8.90	8.90	4.45
Concentration 2 (g/L)	3.16	7.90	0.79	4.45	15.80	15.80	7.90
Concentration 3 (g/L)	5.64	14.10	1.41	7.90	28.10	28.10	14.10
Concentration 4 (g/L) ^b^	10.00	25.00	2.50	14.1	50.00	50.00	25.00
Concentration 5 (g/L)	17.80	44.50	4.45	25.00	88.90	88.90	44.50
Concentration 6 (g/L)	31.60	79.10	7.90	44.50	158.10	158.10	79.10
Compound ^c^	MSG	citrulline	pyroglutamic acid	glutamine	theanine	carnosine	ornithine
Concentration 1 (g/L)	0.90	4.45	0.45	2.25	8.90	8.90	4.45
Concentration 2 (g/L)	1.58	7.90	0.79	4.45	15.80	15.80	7.90
Concentration 3 (g/L)	2.82	14.10	1.41	7.90	28.10	28.10	14.10
Concentration 4 (g/L) ^b^	5.00	25.00	2.50	14.1	50.00	50.00	25.00
Concentration 5 (g/L)	8.90	44.50	4.45	25.00	88.90	88.90	44.50
Concentration 6 (g/L)	15.80	79.10	7.90	44.50	158.10	158.10	79.10
Concentrations for the koku assessment							
Compound	GSH	citrulline	pyroglutamic acid	glutamine	theanine	carnosine	ornithine
Concentration (g/L)	1.5/0.75 ^d^	4.45	0.45	4.45	8.9	8.9	4.45

^a^ HMB means ß-hydroxy-ß methylbutyrate, GABA means γ-aminobutyric acid, MSG means monosodium glutamate, IMP means inosine monophosphate and GSH means reduced glutathione. ^b^ These concentrations were prepared twice, since they were selected as warm-up samples. ^c^ These compounds were diluted in IMP 0.5 mmol/L (0.174 g IMP/L). ^d^ 1.5 g/L was the testing concentration of GSH while 0.75 g/L was selected as the warm-up sample for the koku assessment.

**Table 2:** Koku-related descriptors used in the sensory analysis.

Koku Descriptor	Description	Food Product Examples.
Body thickness ^a^	Perception of viscosity, weight and substantiality in the mouth.	Vegetable cream, whipped cream, etc.
Mouthcoating	Sensation of a layer left in the mouth surfaces after swallowing.	Peanut butter, margarine, etc.
Continuity	An increased duration of the taste after swallowing, making the perception lingers.	Stevia, parmesan cheese, etc.
Astringency	A drying, tightening, or puckering sensation in the mouth.	Red wine, kiwi, red fruits, etc.
Aftertaste	The perception of an increase in the intensity of the food after swallowing.	Parmesan cheese, dry-cured ham, etc.

^a^ The descriptions were adapted from previous publications [26,27].

**Table 3:** In silico evaluation for the 17 compounds obtained using the bioinformatic tool of PredMol (https://hwwlab.com/tastemeta/predmol) [20]. (Accessed on 25 October 2025).

Compound	Sweetness	Saltiness	Sourness	Bitterness	Astringency	Koku	Tasteless
βAla	0.357	0.000	0.038	0.069	0.584	0.002	0.256
Bet	0.027	0.000	0.000	0.641	0.002	0.002	0.115
Car	0.000	0.000	0.000	0.385	0.001	0.999	0.000
Cit	0.070	0.000	0.000	0.036	0.001	0.449	0.189
Cre	0.114	0.000	0.001	0.683	0.002	0.000	0.001
CysCys	0.930	0.000	0.000	0.047	0.001	0.016	0.009
GABA	0.079	0.000	0.037	0.044	0.944	0.007	0.264
Gln	0.542	0.000	0.000	0.031	0.009	0.010	0.012
HMB	0.523	0.000	0.001	0.214	0.237	0.000	0.063
HTrp	0.424	0.000	0.000	0.625	0.001	0.000	0.001
Hyp	0.174	0.000	0.003	0.765	0.002	0.001	0.001
LCar	0.028	0.000	0.000	0.783	0.001	0.000	0.029
Nor	0.976	0.000	0.000	0.018	0.005	0.002	0.001
Orn	0.774	0.000	0.000	0.020	0.009	0.864	0.023
Pyr	0.046	0.000	0.000	0.702	0.000	0.001	0.047
Tau	0.087	0.000	0.000	0.700	0.022	0.010	0.017
The	0.029	0.000	0.000	0.012	0.829	0.966	0.001

**Table 4:** Quantitative descriptive analysis (QDA) for the seventeen compounds expressed in estimated marginal means (emmeans) with the standard error (SE) per each taste attribute.

Compound	Sweetness (SE = 0.64)	Saltiness (SE = 0.53)	Sourness (SE = 0.60)	Bitterness (SE = 0.66)	Umaminess (SE = 0.71)	Pungency (SE = 0.58)	Astringency (SE = 0.67)
βAla	2.98 abcd ^a^	0.48 a	4.87 b	0.51 a	0.89 a	4.12 b	0.81 a
Bet	4.71 def	0.61 a	0.90 a	6.21 efgh	1.66 ab	0.32 a	2.11 ab
Car	6.62 fg	0.44 a	0.87 a	5.06 defgh	1.19 a	0.54 a	0.96 a
Cit	4.04 def	0.14 a	0.34 a	3.55 abcde	2.11 ab	0.48 a	1.26 ab
Cre	1.16 abc	0.54 a	0.75 a	5.36 defgh	2.29 ab	0.71 a	2.39 ab
CysCys	3.44 bcd	0.91 a	1.11 a	3.97 bcdef	1.81 ab	0.38 a	1.17 a
GABA	1.00 ab	0.73 a	5.61 b	1.10 ab	1.29 a	7.64 c	2.03 ab
Gln	3.14 abcd	0.85 a	4.87 b	0.93 ab	4.95 bc	1.26 a	0.86 a
HMB	0.89 ab	1.49 a	1.83 a	7.96 h	0.92 a	1.66 ab	4.31 bc
HTrp	6.56 fg	0.00 a	0.79 a	7.30 gh	1.71 ab	0.95 a	2.54 abc
Hyp	8.29 g	0.00 a	1.73 a	1.86 abc	2.11 ab	0.11 a	1.00 a
LCar	3.96 cdef	1.07 a	1.11 a	5.74 efgh	2.31 ab	1.01 a	1.29 ab
Nor	3.66 bcde	0.66 a	1.90 a	6.81 fgh	1.63 ab	1.22 a	1.63 ab
Orn	2.96 abcd	2.56 a	1.06 a	7.04 fgh	1.04 a	0.35 a	0.88 a
Pyr	0.41 a	2.44 a	9.75 c	2.26 abcd	5.00 bc	2.68 ab	5.62 c
Tau	2.06 abcd	2.27 a	4.98 b	4.25 cdefg	1.43 a	0.79 a	0.96 a
The	6.39 efg	1.54 a	1.21 a	6.00 efgh	6.47 c	1.92 ab	1.36 ab

^a^ Different lowercase letters (a, b, c, d, e, f, g, h) mean significant differences (*p* < 0.05).

## Data Availability

The original contributions presented in this study are included in the article and Supplementary Material. Further inquiries can be directed to the corresponding author.

## Supplementary Materials

The following supporting information can be downloaded at: https://www.mdpi.com/article/10.3390/foods15101826/s1, Table S1. Summary of the different pre-tastings for selecting the concentrations tested in the Quantitative descriptive analysis. Table S2: Pre-tastings of the concentrations tested in the umami and the koku assessment. Table S3. Compilation of slopes and intercepts of umami dose-response curves obtained from the mixed linear model. Figure S1: Summary of chi-square analysis of the variance (ANOVA) for the linear mixed model used in the umami assessment study.
