# Supplementary material for: Comprehensive Taste Profile Assessment of Underexplored Amino Acids and Protein Derivatives in Umami and Koku
Source: Foods. 2026 May 21;15(10):1826. doi: 10.3390/foods15101826 (PMC13205270; doi:10.3390/foods15101826)
Supplement: Supplementary file 1 [file foods-15-01826-s001.zip › foods-4245082-supplementary.pdf]

Supplementary Material

Table S1. Summary of the different pre-tastings for selecting the concentrations tested in the Quantitative descriptive analysis.

Compound	Description
βAla	10g/L: Tasteless. 20g/L: Slight sour. 40g/L: Sweet and sour notable. Slight pungent.
Bet	10g/L: Slight sweet and bitter. 20g/L: Sweet and bitter clearly perceptible.
Car	10g/L: Tasteless. 20g/L: Slight sweet. 40g/L: Sweet and bitter perceptible. Slight sour.
Cit	10g/L: Slight sweet and bitter. 20g/L: Clearly bittersweet. Slight sour and umami.
Cre	10g/L: Clearly bitter and slight sour.
CysCys	10g/L: Clearly bitter and slight sweet.
GABA	10g/L: Tasteless. 20g/L: Slight sour. 40g/L: Pungent and slight sour.
Gln	10g/L: Slight sweet and sour. 20g/L: Clearly sour and sweet. Slight umami.
HMB	10g/L: Bitter and slight sour. 20g/L: Clearly bitter and astringent. Slight sweet and sour.
HTrp	10g/L: Very bitter and very sweet, at the same time.
Hyp	10g/L: Very sweet and slight bitter.
LCar	10g/L: Tasteless. 20g/L: Tasteless. 40g/L: Sweet perceptible 50 g/L: Clearly sweet and slight bitter
Nor	10g/L: Bitter and sweet. 20g/L: Clearly bitter and sweet.
Orn	10g/L: Bitter. 20g/L: Clearly bitter. Slight sweet and umami.
Pyr	10g/L: Very sour, slight umami.
Tau	10g/L: Tasteless. 20g/L: Tasteless. 40g/L: Sour perceptible. 50 g/L: Clearly sour and slight bitter
The	10g/L: Tasteless. 20g/L: Slight sweet and umami. 40g/L: Clearly bitter and umami. Slight sweet.

Table S2: Pre-tastings of the concentrations tested in the umami and the koku assessment.

Concentrations (g/L) for the umami assessment without IMP						
MSG	citrulline	pyroglutamic acid	glutamine	theanine	carnosine	ornithine
0.90 Very slight umami	2.25 Tasteless	0.45 Only sour	2.25 Almost tasteless	4.45 Tasteless	4.45 Tasteless	2.25 Tasteless
1.80 Slight umami	4.45 Very slight umami	0.79 Slight umami	4.45 very slight umami	8.90 Almost tasteless	8.90 Almost tasteless	4.45 Slight umami
3.16 Clearly umami	7.90 slight umami	1.41 Slight umami	7.90 slight umami	15.80 Slight umami	15.80 Almost tasteless	7.90 Notably umami
5.64 Clearly umami	14.10 slight umami	2.50 Notably umami	14.1 clearly umami	28.10 Notably umami	28.10 Very slight umami	14.10 Notably umami
10.00 Very umami	25.00 Umami perceptible	4.45 Clearly umami	25.00 clearly umami	50.00 Clearly umami	50.00 Very slight umami	25.00 Clearly umami
17.80 Very umami	44.50 Clearly umami	7.90 Clearly umami	44.50 Very umami	88.90 Very umami	88.90 Slight umami	44.50 Clearly umami
31.60 extremely umami	79.10 Umami overshadowed by bitterness	14.1 Umami overshadowed by sourness	79.10 Umami overshadowed by sweetness	158.10 Very umami	158.10 Slight umami	79.10 Very umami
Concentrations (g/L) for the umami assessment with IMP						
MSG	citrulline	pyroglutamic acid	glutamine	theanine	carnosine	ornithine
0.45 Slight umami	2.25 Slight umami	0.45 Slight umami	2.25 Almost tasteless	4.45 Almost tasteless	4.45 Almost tasteless	2.25 Slight umami
0.90 Clearly umami	4.45 Slight umami	0.79 Slight umami	4.45 Slight umami	8.90 Slight umami	8.90 Almost tasteless	4.45 Slight umami

1.58 Clearly umami	7.90 Clearly umami	1.41 Notably umami	7.90 Clearly umami	15.80 Slight umami	15.80 Very slight umami	7.90 Notably umami
2.82 Very umami	14.10 Clearly umami	2.50 Notably umami	14.1 clearly umami	28.10 Notably umami	28.10 Very slight umami	14.10 Notably umami
5.00 Very umami	25.00 Very umami	4.45 Clearly umami	25.00 Very umami	50.00 Very umami	50.00 Very slight umami	25.00 Clearly umami
8.90 Extremely umami	44.50 Very umami	7.90 Very umami	44.50 Very umami	88.90 Very umami	88.90 Notably umami	44.50 Very umami
15.80 Reaching the umami saturation	79.10 Umami overshadowed by bitterness	14.1 Umami overshadowed by sourness	79.10 Umami overshadowed by sweetness	158.10 Very umami	158.10 Notably umami	79.10 extremely umami
Concentrations (g/L) for the koku assessment in aqueous solution.						
GSH	citrulline	pyroglutamic acid	glutamine	theanine	carnosine	ornithine
0.25 Koku perceptible.	2.25 No enhancement perceptible	0.45 Clear koku enhacement.	2.25 No enhancement	2.25 Slight koku enhancement.	2.25 No enhancement.	2.25 Slight koku enhancement
0.75 Clearly koku enhacement.	4.45 Clearly koku enhancement. More viscous and savoury.	0.79 Sourness overshadowed koku.	4.45 Slight enhancement perception.	4.45 Slight koku enhancement.	4.45 No enhancement.	4.45 Clear koku enhancement
1.5 Very koku enchacement.	8.9 Bitterness overshadowed koku.	1.41 Sourness overshadowed koku.	7.90 Sourness overshadowed koku.	8.9 Clear koku enhancement. Slight astringent.	8.9 Slight koku enhacement. Perception linger.	8.9 Bitterness overshadowed koku.
Concentrations (g/L) for the koku assessment in mushroom broth.						
GSH	citrulline	pyroglutamic acid	glutamine	theanine	carnosine	ornithine
0.25 Koku perceptible.	2.25 Slight koku enhancement.	0.45 Clear koku enhacement. Increase astringency and continuity.	2.25 No enhancement	2.25 Slight koku enhancement. Increase sweetness.	2.25 No enhancement.	2.25 Slight koku enhancement
0.75 Clearly koku enhacement.	4.45 Clearly koku enhancement. More viscous and increase onion flavours.	0.79 Sourness overshadowed koku.	4.45 Very slight koku enhancement.	4.45 Clear koku enhancement. Increase mushroom flavours.	4.45 Slight koku enhancement.	4.45 Clear koku enhancement Increase onion and garlic flavours.
1.5 Very koku enchacement.	8.9 Bitterness overshadowed koku.	1.41 Sourness overshadowed koku.	7.90 Sourness overshadowed koku.	8.9 Strong koku enhancement. Slight astringent and increase of mushroom flavours	8.9 Slight koku enhacement. Perception linger	8.9 Bitterness overshadowed koku.

Table S3. Compilation of slopes and intercepts of umami dose-response curves obtained from the mixed linear model.

Compound in Water	Slope	intercept	Compound in IMP 0.5 mmol/L	Slope	intercept
MSG	1.22E+01	1.95E+01	MSG	5.24E+00	3.30E+01
carnosine	-9.00E-02	2.95	carnosine	5.80E+00	-6.433
citrulline	9.60E-01	7.49	citrulline	6.87E+00	-1.984
glutamine	1.62E+00	4.74E+00	glutamine	9.73E+00	3.87E+00
ornithine	5.46E+00	8.21E+00	ornithine	7.50E+00	-3.28E-01
pyroglutamic acid	2.47E+00	5.92E+00	pyroglutamic acid	8.29E+00	5.12E+00
theanine	8.60E-01	5.66E+00	theanine	9.17E+00	-7.51E+00

```
Analysis of Deviance Table (Type II Wald chisquare tests)

Response: Umaminess
                             Chisq Df Pr(>Chisq)    
Solution                   67.7249  1  < 2.2e-16 ***
logconc                    40.2123  1  2.278e-10 ***
Aminoacid                 358.9745  6  < 2.2e-16 ***
Bitterness                  0.6863  1    0.40744    
Sweetness                  77.4326  1  < 2.2e-16 ***
Solution:logconc            5.2917  1    0.02143 *  
Solution:Aminoacid         31.9309  6  1.682e-05 ***
logconc:Aminoacid          60.9734  6  2.854e-11 ***
Bitterness:Sweetness        7.5959  1    0.00585 ** 
Solution:logconc:Aminoacid 16.2982  6    0.01224 *  
---
Signif. codes:  0 ‘***’ 0.001 ‘**’ 0.01 ‘*’ 0.05 ‘.’ 0.1 ‘ ’ 1
```

Figure S1. Summary of chi-square analysis of the variance (ANOVA) for the linear mixed model used in the umami assessment study.
